# Supplementary material for: Leaf Traits Explain the Growth Variation and Nitrogen Response of Eucalyptus urophylla × Eucalyptus grandis and Dalbergia odorifera in Mixed Culture
Source: Plants (Basel). 2024 Mar 29;13(7):988. doi: 10.3390/plants13070988 (PMC11013580; doi:10.3390/plants13070988)
Supplement: Supplementary file 1 [file plants-13-00988-s001.zip › plants-2859534-supplementary.pdf]

Table S1. List of leaf functional trait indicators analyzed in this paper and their descriptions.

Leaf functional traits		Abbreviation	Unit	Description
Leaf morphological traits	Leaf area	LA	cm^2	Light capture.
	Leaf thickness	LT	mm	Water use efficiency of leaves; leaf stomatal behavior.
	Leaf fresh mass	LFM	g	Growth strategy and response to environmental variations in leaves.
	Leaf dry mass	LDM	g	Growth strategy and response to environmental variations in leaves.
	Leaf volume	LV	cm^3	Leaf growth and development.
	Specific leaf area	SLA	$cm^2\ g^{-1}$	Light capture, relative growth rate and plant tolerance.
	Leaf tissue density	LTD	$g\ cm^{-3}$	Relates to photosynthesis, resistance and defense.
	Leaf dry matter content	LDMC	$g\ g^{-1}$	The ratio of dry mass to fresh mass; plant resistance.
	Leaf mass fraction	LMF	$g\ g^{-1}$	Biomass of the plant allocated to leaves for light capture.
Leaf physiological traits	Leaf nitrogen content	LNC	$g\ kg^{-1}$	Nitrogen nutrition and photosynthesis in plants.
	Net photosynthetic rate	Pn	$\mu mol\ m^{-2}\ s^{-1}$	Organic matter accumulation and plant growth.
	photosynthetic N use sufficiency	PNUE	$\mu mol\ g^{-1}\ s^{-1}$	The extent to which photosynthesis utilizes nitrogen.
	Stomatal conductance	gs	$mol\ m^{-2}\ s^{-1}$	Balance of carbon uptake and water loss in leaves.
	Transpiration rate	Tr	$\mu mol\ m^{-2}\ s^{-1}$	Water utilization and protection of leaves.
	Intercellular CO_2 concentration	Ci	$\mu mol\ mol^{-1}$	Photosynthetic efficiency; the response to adversity.
	Water use efficiency	WUE	$\mu mol\ mol^{-1}$	Response to adversity.
	Carboxylation efficiency of rubisco	CE	$mol\ m^{-2}\ s^{-1}$	Photosynthetic efficiency.
	Chlorophyll a	Chl a	$mg\ g^{-1}$	Main photosynthetic pigments.
	Chlorophyll b	Chl b	$mg\ g^{-1}$	Main photosynthetic pigments.
	Total amount of leaf chlorophyll content	Chl (a+b)	$mg\ g^{-1}$	Photosynthetic capacity of plants.
	Carotenoid content	Car	$mg\ g^{-1}$	Accessory pigments; photosynthetic system protection.

Table S2. Effects of cultivation pattern, N addition, and their interactions on the leaf traits of *Eucalyptus urophylla* × *E. grandis* and *D. odorifera*.

Species	Leaf traits	Cultivation pattern (C), d.f. =1	Nitrogen addition (N), d.f. =3	C × N, d.f. = 3
		F	***F***	***F***
Eucalyptus urophylla × *E. grandis*	LA	12.90^{***}	7.73^{***}	13.90^{***}
	LT	7.33^{**}	59.04^{***}	0.31^{ns}
	LV	2.42^{ns}	16.00^{***}	10.04^{***}
	LFM	0.13^{ns}	25.49^{***}	2.91^{*}
	LDM	13.15^{***}	9.50^{***}	38.08^{***}
	LDMC	42.17^{***}	49.12^{***}	124.21^{***}
	SLA	0.04^{ns}	12.32^{***}	33.45^{***}
	LTD	9.52^{**}	25.38^{***}	82.32^{***}
	LMF	15.38^{***}	20.55^{***}	0.93^{ns}
	Pn	85.75^{***}	153.52^{***}	0.2^{ns}
	gs	0.62^{ns}	7.72^{**}	0.82^{ns}
	Tr	19.68^{***}	9.35^{***}	2.09^{ns}
	Ci	13.70^{**}	55.24^{***}	1.52^{ns}
	WUE	31.44^{***}	112.47^{***}	5.17^{**}
	CE	58.47^{***}	122.40^{***}	0.93^{ns}
	Chl a	0.88^{ns}	21.09^{***}	2.53^{ns}
	Chl b	0.10^{ns}	24.73^{***}	2.05^{ns}
	Chl a+b	0.97^{ns}	29.94^{***}	2.73^{*}
	Car	1.45^{ns}	37.85^{***}	2.22^{ns}
	LNC	14.31^{**}	175.30^{***}	2.98^{*}
D. odorifera	LA	428.63^{***}	36.76^{***}	7.35^{***}
	LT	220.71^{***}	17.37^{***}	8.55^{***}
	LV	966.05^{***}	30.64^{***}	2.30^{ns}
	LFM	450.89^{***}	25.18^{***}	3.51^{*}
	LDM	415.20^{***}	23.75^{***}	2.56^{ns}
	LDMC	1.72^{ns}	18.98^{***}	11.64^{***}
	SLA	88.08^{***}	7.57^{***}	2.43^{ns}
	LTD	100.38^{***}	21.62^{***}	13.69^{***}
	LMF	44.89^{***}	14.24^{***}	0.55^{ns}
	Pn	455.42^{***}	14.98^{***}	37.39^{***}
	gs	9.04^{**}	29.63^{***}	7.65^{**}
	Tr	24.14^{***}	66.86^{***}	10.71^{***}
	Ci	46.13^{***}	5.02^{**}	6.38^{**}
	WUE	104.16^{***}	31.55^{***}	2.83^{*}
	CE	362.38^{***}	19.04^{***}	35.13^{***}
	Chl a	59.71^{***}	1.78^{ns}	5.40^{**}
	Chl b	42.40^{***}	5.75^{***}	4.25^{**}
	Chl a+b	62.84^{***}	2.72^{*}	4.86^{**}
	Car	31.10^{***}	1.56^{ns}	3.79^{*}
	LNC	5.81^{*}	12.88^{***}	4.94^{**}

The symbols "*, ** and ***" indicate significant differences at the 0.05, 0.01 and 0.001 levels, respectively. Nonsignificant differences (P > 0.05) between treatments are indicated by "ns".

Table S3. The r values and p values of Pearson's correlation analysis of leaf traits of *E. urophylla* × *E. grandis* in monoculture.

Leaf traits	LA	LT	LV	LFM	LDM	LDMC	SLA	LTD	LMF	Pn	gs	Tr	Ci	WUE	CE	LNC	PNUE	Chl a	Chl b	Chl a+b	Car
LA	−	−0.527	0.533	−0.064	0.315	0.371	0.322	−0.039	−0.245	0.226	0.226	0.415	−0.324	0.151	0.249	0.248	0.100	−0.066	0.039	−0.032	−0.011
LT	0.017	−	0.428	0.621	0.143	−0.342	−0.473	−0.128	0.293	−0.522	−0.206	−0.544	0.494	−0.444	−0.502	−0.488	−0.405	−0.396	−0.459	−0.425	−0.396
LV	0.015	0.060	−	0.574	0.466	0.026	−0.129	−0.178	0.072	−0.329	−0.029	−0.137	0.189	−0.327	−0.286	−0.259	−0.322	−0.499	−0.443	−0.491	−0.450
LFM	0.789	0.004	0.008	−	0.390	−0.395	−0.471	0.053	0.587	−0.529	−0.296	−0.649	0.483	−0.436	−0.524	−0.467	−0.509	−0.588	−0.688	−0.634	−0.633
LDM	0.176	0.547	0.038	0.089	−	0.689	−0.782	0.783	0.080	0.103	−0.164	−0.238	−0.168	0.185	0.092	0.245	−0.083	−0.169	−0.217	−0.188	0.020
LDMC	0.107	0.140	0.915	0.085	0.001	−	−0.405	0.731	−0.390	0.524	0.063	0.264	−0.551	0.533	0.512	0.611	0.319	0.303	0.332	0.320	0.529
SLA	0.167	0.035	0.588	0.036	0.000	0.077	−	−0.798	−0.265	0.038	0.318	0.485	−0.032	−0.087	0.057	−0.087	0.121	0.134	0.271	0.182	−0.001
LTD	0.869	0.589	0.452	0.825	0.000	0.000	0.000	−	0.068	0.330	−0.182	−0.177	−0.306	0.421	0.288	0.446	0.135	0.147	0.045	0.117	0.316
LMF	0.299	0.209	0.762	0.007	0.739	0.089	0.259	0.777	−	−0.739	−0.657	−0.541	0.734	−0.693	−0.772	−0.619	−0.754	−0.688	−0.748	−0.722	−0.731
Pn	0.339	0.018	0.157	0.016	0.666	0.018	0.874	0.155	0.000	−	0.699	0.568	−0.913	0.979	0.987	0.921	0.839	0.838	0.744	0.823	0.887
gs	0.338	0.383	0.905	0.205	0.490	0.791	0.172	0.442	0.002	0.001	−	0.499	−0.633	0.668	0.692	0.558	0.692	0.449	0.368	0.431	0.465
Tr	0.069	0.013	0.564	0.002	0.311	0.261	0.030	0.455	0.014	0.009	0.025	−	−0.633	0.395	0.604	0.461	0.449	0.432	0.493	0.461	0.438
Ci	0.163	0.027	0.425	0.031	0.479	0.012	0.892	0.189	0.000	0.000	0.003	0.003	−	−0.871	−0.948	−0.881	−0.742	−0.724	−0.623	−0.705	−0.814
WUE	0.526	0.050	0.160	0.054	0.435	0.016	0.716	0.065	0.001	0.000	0.001	0.085	0.000	−	0.953	0.923	0.835	0.825	0.706	0.802	0.883
CE	0.289	0.024	0.221	0.018	0.700	0.021	0.811	0.219	0.000	0.000	0.001	0.005	0.000	0.000	−	0.910	0.824	0.829	0.725	0.811	0.884
LNC	0.291	0.029	0.270	0.038	0.298	0.004	0.714	0.049	0.004	0.000	0.011	0.041	0.000	0.000	0.000	−	0.692	0.758	0.656	0.740	0.837
PNUE	0.674	0.076	0.166	0.022	0.729	0.171	0.611	0.571	0.000	0.000	0.001	0.047	0.000	0.000	0.000	0.001	−	0.748	0.661	0.734	0.730
Chl a	0.782	0.084	0.025	0.006	0.475	0.193	0.574	0.535	0.001	0.000	0.047	0.057	0.000	0.000	0.000	0.000	0.000	−	0.911	0.990	0.949
Chl b	0.871	0.042	0.050	0.001	0.357	0.153	0.248	0.849	0.000	0.000	0.110	0.027	0.003	0.001	0.000	0.002	0.002	0.000	−	0.959	0.863
Chl a+b	0.892	0.062	0.028	0.003	0.426	0.170	0.442	0.624	0.000	0.000	0.058	0.041	0.001	0.000	0.000	0.000	0.000	0.000	0.000	−	0.939
Car	0.963	0.084	0.047	0.003	0.933	0.016	0.997	0.175	0.000	0.000	0.039	0.053	0.000	0.000	0.000	0.000	0.000	0.000	0.000	0.000	−

The data in the upper right corner indicate r-values and the data in the lower left corner indicate p–values.

Table S4. The t values and standard error of Pearson's correlation analysis of leaf traits of *E. urophylla* × *E. grandis* in monoculture.

Leaf traits	LA	LT	LV	LFM	LDM	LDMC	SLA	LTD	LMF	Pn	gs	Tr	Ci	WUE	CE	LNC	PNUE	Chl a	Chl b	Chl a+b	Car
LA	−	−2.633	2.674	−0.271	1.409	1.697	1.442	−0.167	−1.070	0.983	0.984	1.935	−1.455	0.647	1.092	1.088	0.428	−0.281	0.164	−0.138	−0.047
LT	0.200	−	2.009	3.358	0.614	−1.544	−2.279	−0.549	1.302	−2.600	−0.894	−2.752	2.409	−2.102	−2.462	−2.375	−1.881	−1.828	−2.191	−1.990	−1.828
LV	0.199	0.213	−	2.975	2.233	0.108	−0.551	−0.768	0.308	−1.476	−0.121	−0.588	0.816	−1.467	−1.267	−1.137	−1.443	−2.446	−2.096	−2.389	−2.137
LFM	0.235	0.185	0.193	−	1.796	−1.823	−2.265	0.225	3.076	−2.648	−1.316	−3.620	2.342	−2.057	−2.611	−2.241	−2.511	−3.088	−4.022	−3.475	−3.472
LDM	0.224	0.233	0.209	0.217	−	4.036	−5.326	5.344	0.339	0.439	−0.705	−1.041	−0.723	0.799	0.391	1.071	−0.351	−0.730	−0.945	−0.814	0.085
LDMC	0.219	0.221	0.236	0.217	0.171	−	−1.879	4.548	−1.795	2.607	0.269	1.161	−2.801	2.672	2.527	3.276	1.427	1.351	1.494	1.431	2.648
SLA	0.223	0.208	0.234	0.208	0.147	0.216	−	−5.625	−1.166	0.161	1.422	2.352	−0.138	−0.369	0.243	−0.372	0.518	0.573	1.195	0.785	−0.004
LTD	0.236	0.234	0.232	0.235	0.147	0.161	0.142	−	0.288	1.483	−0.786	−0.763	−1.365	1.968	1.274	2.116	0.577	0.632	0.193	0.498	1.413
LMF	0.229	0.225	0.235	0.191	0.235	0.217	0.227	0.235	−	−4.660	−3.702	−2.729	4.589	−4.075	−5.159	−3.347	−4.868	−4.024	−4.781	−4.430	−4.549
Pn	0.230	0.201	0.223	0.200	0.234	0.201	0.236	0.222	0.159	−	4.151	2.931	−9.490	20.442	25.801	10.064	6.552	6.513	4.718	6.152	8.137
gs	0.230	0.231	0.236	0.225	0.233	0.235	0.223	0.232	0.178	0.168	−	2.446	−3.468	3.811	4.065	2.855	4.068	2.134	1.681	2.026	2.226
Tr	0.214	0.198	0.233	0.179	0.229	0.227	0.206	0.232	0.198	0.194	0.204	−	−3.471	1.825	3.217	2.202	2.133	2.033	2.405	2.205	2.067
Ci	0.223	0.205	0.231	0.206	0.232	0.197	0.236	0.224	0.160	0.096	0.182	0.182	−	−7.509	−12.659	−7.913	−4.703	−4.457	−3.377	−4.220	−5.947
WUE	0.233	0.211	0.223	0.212	0.232	0.199	0.235	0.214	0.170	0.048	0.175	0.217	0.116	−	13.293	10.177	6.440	6.192	4.227	5.691	7.963
CE	0.228	0.204	0.226	0.201	0.235	0.203	0.235	0.226	0.150	0.038	0.170	0.188	0.075	0.072	−	9.317	6.173	6.298	4.463	5.885	8.018
LNC	0.228	0.206	0.228	0.208	0.229	0.187	0.235	0.211	0.185	0.092	0.196	0.209	0.111	0.091	0.098	−	4.064	4.936	3.690	4.666	6.480
PNUE	0.235	0.215	0.223	0.203	0.235	0.223	0.234	0.234	0.155	0.128	0.170	0.211	0.158	0.130	0.133	0.170	−	4.787	3.734	4.586	4.526
Chl a	0.235	0.216	0.204	0.191	0.232	0.225	0.234	0.233	0.171	0.129	0.211	0.213	0.163	0.133	0.132	0.154	0.156	−	9.361	30.336	12.728
Chl b	0.236	0.209	0.211	0.171	0.230	0.222	0.227	0.235	0.156	0.158	0.219	0.205	0.184	0.167	0.162	0.178	0.177	0.097	−	14.397	7.252
Chl a+b	0.236	0.213	0.205	0.182	0.231	0.223	0.232	0.234	0.163	0.134	0.213	0.209	0.167	0.141	0.138	0.159	0.160	0.033	0.067	−	11.624
Car	0.236	0.216	0.211	0.182	0.236	0.200	0.236	0.224	0.161	0.109	0.209	0.212	0.137	0.111	0.110	0.129	0.161	0.075	0.119	0.081	−

The data in the upper right corner indicate t-values and the data in the lower left corner indicate standard error.

Table S5. The r values and p values of Pearson's correlation analysis of leaf traits of *E. urophylla* × *E. grandis* in mixed culture.

Leaf traits	LA	LT	LV	LFM	LDM	LDMC	SLA	LTD	LMF	Pn	gs	Tr	Ci	WUE	CE	LNC	PNUE	Chl a	Chl b	Chl a+b	Car
LA	−	−0.183	0.757	0.678	0.636	0.148	−0.027	0.305	0.400	−0.152	0.092	−0.260	0.005	−0.033	−0.117	−0.071	−0.202	−0.156	−0.044	−0.105	−0.074
LT	0.440	−	0.499	0.515	0.562	0.179	−0.887	0.466	0.398	−0.325	−0.244	−0.319	0.371	−0.283	−0.285	−0.415	0.145	−0.383	−0.381	−0.420	−0.486
LV	0.000	0.025	−	0.935	0.935	0.262	−0.610	0.578	0.589	−0.323	−0.040	−0.418	0.224	−0.195	−0.262	−0.308	−0.078	−0.374	−0.259	−0.343	−0.358
LFM	0.001	0.020	0.000	−	0.909	0.044	−0.627	0.619	0.625	−0.317	0.006	−0.447	0.212	−0.168	−0.253	−0.325	−0.077	−0.393	−0.331	−0.386	−0.419
LDM	0.003	0.010	0.000	0.000	−	0.452	−0.773	0.826	0.678	−0.418	−0.100	−0.481	0.316	−0.302	−0.357	−0.418	−0.176	−0.453	−0.354	−0.399	−0.362
LDMC	0.534	0.450	0.265	0.855	0.046	−	−0.465	0.635	0.323	−0.341	−0.256	−0.187	0.298	−0.387	−0.325	−0.309	−0.234	−0.231	−0.142	−0.129	0.047
SLA	0.911	0.000	0.004	0.003	0.000	0.039	−	−0.804	−0.603	0.429	0.280	0.399	−0.416	0.376	0.382	0.486	0.042	0.515	0.484	0.496	0.464
LTD	0.191	0.038	0.008	0.004	0.000	0.003	0.000	−	0.665	−0.469	−0.192	−0.457	0.370	−0.399	−0.418	−0.476	−0.295	−0.461	−0.410	−0.385	−0.262
LMF	0.080	0.083	0.006	0.003	0.001	0.165	0.005	0.001	−	−0.814	−0.535	−0.697	0.642	−0.700	−0.763	−0.786	−0.098	−0.635	−0.723	−0.689	−0.633
Pn	0.523	0.162	0.165	0.173	0.067	0.141	0.059	0.037	0.000	−	0.689	0.790	−0.867	0.937	0.965	0.910	0.058	0.422	0.609	0.522	0.482
gs	0.698	0.301	0.866	0.982	0.676	0.276	0.232	0.417	0.015	0.001	−	0.451	−0.570	0.695	0.664	0.647	−0.005	0.200	0.328	0.281	0.269
Tr	0.268	0.171	0.067	0.048	0.032	0.429	0.082	0.043	0.001	0.000	0.046	−	−0.686	0.535	0.756	0.655	0.279	0.386	0.421	0.384	0.393
Ci	0.984	0.107	0.343	0.369	0.174	0.202	0.068	0.108	0.002	0.000	0.009	0.001	−	−0.819	−0.947	−0.874	0.100	−0.371	−0.570	−0.496	−0.461
WUE	0.890	0.227	0.409	0.480	0.196	0.092	0.102	0.081	0.001	0.000	0.001	0.015	0.000	−	0.903	0.887	−0.078	0.341	0.570	0.473	0.410
CE	0.624	0.224	0.265	0.282	0.123	0.162	0.097	0.067	0.000	0.000	0.001	0.000	0.000	0.000	−	0.899	0.044	0.419	0.608	0.524	0.487
LNC	0.766	0.069	0.186	0.162	0.067	0.185	0.030	0.034	0.000	0.000	0.002	0.002	0.000	0.000	0.000	−	−0.087	0.386	0.623	0.523	0.493
PNUE	0.394	0.541	0.745	0.748	0.459	0.322	0.859	0.207	0.682	0.809	0.983	0.234	0.675	0.744	0.854	0.714	−	0.358	0.092	0.151	0.156
Chl a	0.511	0.095	0.104	0.087	0.045	0.327	0.020	0.041	0.003	0.064	0.397	0.093	0.107	0.141	0.066	0.093	0.121	−	0.847	0.949	0.900
Chl b	0.853	0.097	0.270	0.154	0.126	0.550	0.031	0.073	0.000	0.004	0.158	0.064	0.009	0.009	0.004	0.003	0.698	0.000	−	0.939	0.881
Chl a+b	0.658	0.065	0.139	0.093	0.082	0.588	0.026	0.094	0.001	0.018	0.231	0.095	0.026	0.035	0.018	0.018	0.525	0.000	0.000	−	0.958
Car	0.756	0.030	0.121	0.066	0.116	0.843	0.039	0.265	0.003	0.032	0.251	0.087	0.041	0.073	0.029	0.027	0.512	0.000	0.000	0.000	−

The data in the upper right corner indicate r-values and the data in the lower left corner indicate p–values.

Table S6. The t values and standard error of Pearson's correlation analysis of leaf traits of *E. urophylla* × *E. grandis* in mixed culture.

Leaf traits	LA	LT	LV	LFM	LDM	LDMC	SLA	LTD	LMF	Pn	gs	Tr	Ci	WUE	CE	LNC	PNUE	Chl a	Chl b	Chl a+b	Car
LA	–	−0.790	4.915	3.915	3.495	0.634	−0.113	1.359	1.854	−0.651	0.394	−1.144	0.021	−0.141	−0.498	−0.303	−0.873	−0.670	−0.188	−0.450	−0.315
LT	0.232	–	2.441	2.547	2.881	0.773	−8.162	2.236	1.838	−1.459	−1.065	−1.426	1.694	−1.251	−1.259	−1.934	0.623	−1.761	−1.749	−1.964	−2.359
LV	0.154	0.204	–	11.172	11.222	1.151	−3.264	3.003	3.095	−1.446	−0.171	−1.951	0.973	−0.845	−1.150	−1.375	−0.331	−1.713	−1.137	−1.548	−1.629
LFM	0.173	0.202	0.084	–	9.231	0.185	−3.418	3.346	3.396	−1.418	0.023	−2.122	0.921	−0.721	−1.110	−1.457	−0.327	−1.813	−1.487	−1.774	−1.960
LDM	0.182	0.195	0.083	0.098	–	2.148	−5.173	6.214	3.917	−1.951	−0.425	−2.326	1.414	−1.342	−1.620	−1.953	−0.757	−2.157	−1.606	−1.843	−1.650
LDMC	0.233	0.232	0.227	0.235	0.210	–	−2.228	3.489	1.447	−1.539	−1.124	−0.809	1.324	−1.780	−1.457	−1.377	−1.019	−1.007	−0.609	−0.552	0.201
SLA	0.236	0.109	0.187	0.184	0.149	0.209	–	−5.731	−3.203	2.013	1.236	1.845	−1.942	1.723	1.753	2.360	0.180	2.549	2.346	2.425	2.224
LTD	0.224	0.209	0.192	0.185	0.133	0.182	0.140	–	3.777	−2.250	−0.830	−2.177	1.689	−1.849	−1.950	−2.294	−1.308	−2.203	−1.905	−1.771	−1.152
LMF	0.216	0.216	0.190	0.184	0.173	0.223	0.188	0.176	–	−5.938	−2.688	−4.125	3.551	−4.156	−5.016	−5.387	−0.416	−3.489	−4.436	−4.034	−3.471
Pn	0.233	0.223	0.223	0.224	0.214	0.222	0.213	0.208	0.137	–	4.028	5.468	−7.390	11.426	15.652	9.292	0.246	1.976	3.253	2.593	2.332
gs	0.235	0.229	0.236	0.236	0.235	0.228	0.226	0.231	0.199	0.171	–	2.144	−2.946	4.099	3.765	3.601	−0.022	0.868	1.472	1.240	1.187
Tr	0.228	0.223	0.214	0.211	0.207	0.232	0.216	0.210	0.169	0.144	0.210	–	−3.999	2.690	4.907	3.673	1.232	1.773	1.972	1.765	1.812
Ci	0.236	0.219	0.230	0.230	0.224	0.225	0.214	0.219	0.181	0.117	0.194	0.172	–	−6.054	−12.530	−7.630	0.426	−1.694	−2.942	−2.424	−2.203
WUE	0.236	0.226	0.231	0.232	0.225	0.217	0.218	0.216	0.168	0.082	0.170	0.199	0.135	–	8.924	8.170	−0.332	1.540	2.947	2.278	1.907
CE	0.234	0.226	0.227	0.228	0.220	0.223	0.218	0.214	0.152	0.062	0.176	0.154	0.076	0.101	–	8.733	0.187	1.958	3.253	2.611	2.367
LNC	0.235	0.214	0.224	0.223	0.214	0.224	0.206	0.207	0.146	0.098	0.180	0.178	0.115	0.109	0.103	–	−0.372	1.775	3.377	2.602	2.407
PNUE	0.231	0.233	0.235	0.235	0.232	0.229	0.235	0.225	0.235	0.235	0.236	0.226	0.235	0.235	0.235	0.235	–	1.625	0.394	0.648	0.669
Chl a	0.233	0.218	0.219	0.217	0.210	0.229	0.202	0.209	0.182	0.214	0.231	0.217	0.219	0.222	0.214	0.217	0.220	–	6.750	12.776	8.738
Chl b	0.235	0.218	0.228	0.222	0.220	0.233	0.206	0.215	0.163	0.187	0.223	0.214	0.194	0.194	0.187	0.184	0.235	0.125	–	11.628	7.903
Chl a+b	0.234	0.214	0.221	0.217	0.216	0.234	0.205	0.218	0.171	0.201	0.226	0.218	0.205	0.208	0.201	0.201	0.233	0.074	0.081	–	14.190
Car	0.235	0.206	0.220	0.214	0.220	0.235	0.209	0.227	0.182	0.207	0.227	0.217	0.209	0.215	0.206	0.205	0.233	0.103	0.111	0.068	–

The data in the upper right corner indicate t-values and the data in the lower left corner indicate standard error.

Table S7. The r values and p values of Pearson's correlation analysis of leaf traits of *D. odorifera* in monoculture.

Leaf traits	LA	LT	LV	LFM	LDM	LDMC	SLA	LTD	LMF	Pn	gs	Tr	Ci	WUE	CE	LNC	PNUE	Chl a	Chl b	Chl a+b	Car
LA	−	0.191	0.781	0.869	0.808	0.030	−0.087	0.129	0.143	−0.278	−0.265	−0.087	−0.016	−0.276	−0.216	−0.105	−0.182	−0.109	−0.162	−0.140	−0.077
LT	0.421	−	0.022	0.415	0.129	−0.388	−0.033	0.099	−0.482	−0.087	−0.064	−0.230	0.022	0.106	−0.072	−0.216	−0.021	0.217	0.208	0.254	0.313
LV	0.000	0.927	−	0.664	0.698	0.143	−0.102	−0.274	0.200	−0.464	−0.416	−0.201	0.120	−0.220	−0.384	−0.137	−0.424	−0.337	−0.403	−0.414	−0.262
LFM	0.000	0.069	0.001	−	0.892	−0.001	−0.351	0.346	0.137	−0.232	−0.185	−0.181	−0.070	−0.090	−0.165	−0.146	−0.264	−0.156	−0.189	−0.173	−0.088
LDM	0.000	0.587	0.001	0.000	−	0.420	−0.623	0.482	0.438	−0.304	−0.270	−0.172	0.021	−0.084	−0.242	−0.164	−0.268	−0.259	−0.256	−0.303	−0.196
LDMC	0.899	0.091	0.548	0.998	0.065	−	−0.759	0.468	0.613	−0.238	−0.247	−0.011	0.201	−0.070	−0.234	−0.097	−0.100	−0.184	−0.046	−0.230	−0.041
SLA	0.717	0.889	0.668	0.130	0.003	0.000	−	−0.734	−0.541	0.163	0.125	0.129	−0.078	−0.143	0.149	0.162	0.147	0.185	0.087	0.216	0.093
LTD	0.586	0.679	0.242	0.135	0.031	0.038	0.000	−	0.360	0.063	0.053	−0.047	−0.055	0.168	0.052	−0.104	0.083	0.026	0.143	0.059	0.025
LMF	0.548	0.032	0.398	0.565	0.053	0.004	0.014	0.119	−	−0.030	0.033	0.107	0.226	−0.063	−0.086	0.049	0.119	−0.185	−0.365	−0.274	−0.209
Pn	0.235	0.714	0.039	0.324	0.193	0.313	0.493	0.790	0.900	−	0.895	0.762	−0.741	−0.187	0.980	0.762	0.849	0.403	0.362	0.429	0.321
gs	0.259	0.790	0.068	0.435	0.249	0.294	0.601	0.823	0.890	0.000	−	0.867	−0.666	−0.447	0.864	0.801	0.763	0.324	0.289	0.354	0.253
Tr	0.716	0.330	0.396	0.445	0.467	0.965	0.589	0.843	0.655	0.000	0.000	−	−0.528	−0.741	0.714	0.818	0.763	0.393	0.383	0.391	0.407
Ci	0.946	0.926	0.614	0.768	0.931	0.395	0.744	0.819	0.337	0.000	0.001	0.017	−	0.160	−0.849	−0.573	−0.446	−0.048	−0.146	−0.106	−0.028
WUE	0.239	0.657	0.352	0.707	0.724	0.770	0.547	0.479	0.791	0.430	0.048	0.000	0.500	−	−0.160	−0.469	−0.319	−0.243	−0.281	−0.230	−0.334
CE	0.360	0.763	0.095	0.486	0.305	0.320	0.530	0.827	0.720	0.000	0.000	0.000	0.000	0.500	−	0.750	0.769	0.314	0.301	0.345	0.243
LNC	0.658	0.360	0.565	0.538	0.489	0.683	0.496	0.662	0.839	0.000	0.000	0.000	0.008	0.037	0.000	−	0.612	0.151	0.102	0.164	0.109
PNUE	0.441	0.930	0.063	0.261	0.253	0.674	0.536	0.729	0.617	0.000	0.000	0.000	0.049	0.170	0.000	0.004	−	0.521	0.433	0.526	0.436
Chl a	0.646	0.359	0.146	0.512	0.270	0.436	0.434	0.913	0.436	0.078	0.164	0.086	0.839	0.301	0.178	0.525	0.018	−	0.713	0.979	0.789
Chl b	0.494	0.380	0.078	0.425	0.277	0.847	0.714	0.548	0.114	0.117	0.216	0.095	0.538	0.230	0.197	0.668	0.056	0.000	−	0.759	0.797
Chl a+b	0.557	0.279	0.070	0.467	0.194	0.330	0.359	0.804	0.243	0.059	0.126	0.088	0.658	0.329	0.137	0.491	0.017	0.000	0.000	−	0.770
Car	0.748	0.179	0.264	0.712	0.408	0.863	0.695	0.918	0.377	0.168	0.282	0.075	0.908	0.150	0.301	0.648	0.055	0.000	0.000	0.000	−

The data in the upper right corner indicate r-values and the data in the lower left corner indicate p−values.

Table S8. The t values and standard error of Pearson's correlation analysis of leaf traits of *D. odorifera* in monoculture.

Leaf traits	LA	LT	LV	LFM	LDM	LDMC	SLA	LTD	LMF	Pn	gs	Tr	Ci	WUE	CE	LNC	PNUE	Chl a	Chl b	Chl a+b	Car
LA		0.823	5.300	7.434	5.818	0.129	−0.369	0.554	0.612	−1.228	−1.166	−0.370	−0.069	−1.219	−0.939	−0.450	−0.787	−0.467	−0.698	−0.598	−0.326
LT	0.231	–	0.093	1.937	0.554	−1.789	−0.142	0.420	−2.332	−0.372	−0.270	−1.002	0.094	0.451	−0.306	−0.939	−0.089	0.942	0.901	1.116	1.399
LV	0.147	0.236	–	3.772	4.131	0.613	−0.435	−1.209	0.865	−2.224	−1.942	−0.869	0.514	−0.956	−1.764	−0.587	−1.984	−1.520	−1.869	−1.929	−1.152
LFM	0.117	0.214	0.176	–	8.369	−0.003	−1.589	1.565	0.586	−1.014	−0.798	−0.781	−0.299	−0.382	−0.711	−0.627	−1.161	−0.669	−0.816	−0.743	−0.376
LDM	0.139	0.234	0.169	0.107	–	1.964	−3.382	2.335	2.070	−1.354	−1.190	−0.742	0.088	−0.359	−1.057	−0.706	−1.181	−1.139	−1.122	−1.348	−0.847
LDMC	0.236	0.217	0.233	0.236	0.214	–	−4.948	2.244	3.290	−1.039	−1.080	−0.045	0.872	−0.297	−1.023	−0.415	−0.428	−0.796	−0.195	−1.002	−0.175
SLA	0.235	0.236	0.234	0.221	0.184	0.153	–	−4.585	−2.729	0.699	0.533	0.550	−0.332	−0.614	0.641	0.695	0.630	0.800	0.372	0.941	0.398
LTD	0.234	0.235	0.227	0.221	0.206	0.208	0.160	–	1.636	0.270	0.227	−0.201	−0.232	0.723	0.222	−0.445	0.352	0.111	0.612	0.251	0.105
LMF	0.233	0.207	0.231	0.233	0.212	0.186	0.198	0.220	–	−0.128	0.140	0.454	0.985	−0.269	−0.364	0.207	0.508	−0.797	−1.662	−1.207	−0.907
Pn	0.226	0.235	0.209	0.229	0.225	0.229	0.233	0.235	0.236	–	8.536	4.993	−4.688	−0.808	20.808	4.992	6.809	1.868	1.649	2.016	1.436
gs	0.227	0.235	0.214	0.232	0.227	0.228	0.234	0.235	0.236	0.105	–	7.394	−3.786	−2.120	7.291	5.681	5.012	1.452	1.281	1.605	1.109
Tr	0.235	0.229	0.231	0.232	0.232	0.236	0.234	0.235	0.234	0.153	0.117	–	−2.638	−4.686	4.332	6.037	5.012	1.814	1.760	1.802	1.891
Ci	0.236	0.236	0.234	0.235	0.236	0.231	0.235	0.235	0.230	0.158	0.176	0.200	–	0.689	−6.831	−2.964	−2.112	−0.206	−0.628	−0.450	−0.117
WUE	0.227	0.234	0.230	0.235	0.235	0.235	0.233	0.232	0.235	0.232	0.211	0.158	0.233	–	−0.688	−2.252	−1.430	−1.065	−1.242	−1.003	−1.502
CE	0.230	0.235	0.218	0.232	0.229	0.229	0.233	0.235	0.235	0.047	0.119	0.165	0.124	0.233	–	4.813	5.101	1.402	1.340	1.558	1.064
LNC	0.234	0.230	0.233	0.233	0.233	0.235	0.233	0.234	0.235	0.153	0.141	0.136	0.193	0.208	0.156	–	3.287	0.648	0.436	0.704	0.465
PNUE	0.232	0.236	0.214	0.227	0.227	0.235	0.233	0.235	0.234	0.125	0.152	0.152	0.211	0.223	0.151	0.186	–	2.592	2.039	2.623	2.056
Chl a	0.234	0.230	0.222	0.233	0.228	0.232	0.232	0.236	0.232	0.216	0.223	0.217	0.235	0.229	0.224	0.233	0.201	–	4.317	20.489	5.452
Chl b	0.233	0.231	0.216	0.231	0.228	0.235	0.235	0.233	0.219	0.220	0.226	0.218	0.233	0.226	0.225	0.234	0.212	0.165	–	4.939	5.593
Chl a+b	0.233	0.228	0.215	0.232	0.225	0.229	0.230	0.235	0.227	0.213	0.220	0.217	0.234	0.229	0.221	0.233	0.200	0.048	0.154	–	5.120
Car	0.235	0.224	0.227	0.235	0.231	0.236	0.235	0.236	0.230	0.223	0.228	0.215	0.236	0.222	0.229	0.234	0.212	0.145	0.142	0.150	–

The data in the upper right corner indicate t-values and the data in the lower left corner indicate standard error.

Table S9. The r values and p values of Pearson's correlation analysis of leaf traits of *D. odorifera* in mixed culture.

Leaf traits	LA	LT	LV	LFM	LDM	LDMC	SLA	LTD	LMF	Pn	gs	Tr	Ci	WUE	CE	LNC	PNUE	Chl a	Chl b	Chl a+b	Car
LA	−	0.129	0.856	0.944	0.916	−0.053	−0.129	0.207	0.184	0.031	−0.232	0.032	0.224	−0.061	−0.017	−0.061	0.028	0.039	−0.111	0.172	0.099
LT	0.588	−	0.589	0.312	0.247	−0.019	−0.512	−0.525	−0.027	0.126	0.027	−0.165	−0.188	0.250	0.136	−0.164	0.317	−0.054	−0.032	−0.052	−0.056
LV	0.000	0.006	−	0.900	0.831	−0.077	−0.296	−0.196	0.204	0.164	−0.169	−0.015	0.114	0.091	0.122	−0.126	0.203	0.036	−0.091	0.148	0.092
LFM	0.000	0.181	0.000	−	0.928	−0.166	−0.269	0.194	0.202	0.157	−0.218	0.003	0.069	0.058	0.121	−0.176	0.160	−0.043	−0.168	0.079	0.065
LDM	0.000	0.294	0.000	0.000	−	0.198	−0.488	0.334	0.157	−0.049	−0.222	0.058	0.152	−0.105	−0.079	−0.207	−0.039	0.034	−0.080	0.173	0.137
LDMC	0.825	0.936	0.746	0.485	0.402	−	−0.627	0.189	−0.098	−0.518	0.019	0.161	0.206	−0.420	−0.498	−0.118	−0.525	0.196	0.255	0.243	0.158
SLA	0.588	0.021	0.205	0.251	0.029	0.003	−	−0.195	−0.031	0.223	0.092	0.028	0.129	0.079	0.183	0.363	0.128	0.092	−0.017	0.036	0.021
LTD	0.381	0.018	0.407	0.412	0.151	0.424	0.411	−	−0.274	−0.270	−0.025	0.125	−0.027	−0.263	−0.250	−0.155	−0.301	−0.007	0.006	0.024	0.106
LMF	0.437	0.910	0.388	0.393	0.510	0.681	0.897	0.242	−	0.077	−0.326	−0.160	0.131	0.041	0.057	−0.054	0.012	−0.357	−0.499	−0.293	−0.328
Pn	0.896	0.596	0.489	0.507	0.839	0.019	0.345	0.250	0.746	−	−0.235	−0.316	−0.422	0.807	0.990	−0.062	0.847	0.190	−0.020	0.117	0.048
gs	0.325	0.911	0.476	0.356	0.346	0.937	0.699	0.918	0.160	0.320	−	0.689	−0.025	−0.413	−0.223	0.429	−0.331	0.185	0.365	0.206	0.315
Tr	0.894	0.486	0.950	0.990	0.807	0.498	0.908	0.599	0.501	0.175	0.001	−	0.195	−0.766	−0.337	0.426	−0.641	0.316	0.489	0.280	0.391
Ci	0.342	0.427	0.631	0.773	0.522	0.384	0.587	0.911	0.582	0.064	0.916	0.409	−	−0.460	−0.533	−0.016	−0.386	−0.230	−0.158	−0.205	−0.198
WUE	0.798	0.289	0.703	0.809	0.659	0.065	0.741	0.263	0.865	0.000	0.070	0.000	0.041	−	0.828	−0.261	0.942	−0.065	−0.247	−0.069	−0.163
CE	0.944	0.567	0.610	0.613	0.740	0.025	0.439	0.288	0.813	0.000	0.344	0.146	0.015	0.000	−	−0.082	0.840	0.184	−0.014	0.118	0.047
LNC	0.798	0.490	0.597	0.457	0.381	0.622	0.116	0.515	0.823	0.794	0.059	0.061	0.945	0.266	0.732	−	−0.088	0.546	0.495	0.400	0.408
PNUE	0.905	0.173	0.391	0.500	0.870	0.017	0.591	0.197	0.961	0.000	0.155	0.002	0.093	0.000	0.000	0.711	−	0.057	−0.135	0.040	−0.020
Chl a	0.870	0.823	0.881	0.856	0.887	0.406	0.698	0.975	0.122	0.422	0.436	0.175	0.330	0.787	0.437	0.013	0.813	−	0.781	0.868	0.769
Chl b	0.641	0.894	0.703	0.478	0.737	0.278	0.945	0.981	0.025	0.934	0.114	0.029	0.505	0.294	0.954	0.027	0.569	0.000	−	0.653	0.603
Chl a+b	0.468	0.827	0.534	0.740	0.466	0.302	0.879	0.920	0.210	0.624	0.384	0.231	0.387	0.774	0.621	0.080	0.866	0.000	0.002	−	0.917
Car	0.678	0.815	0.700	0.786	0.565	0.505	0.931	0.658	0.158	0.840	0.175	0.089	0.403	0.492	0.845	0.074	0.932	0.000	0.005	0.000	−

The data in the upper right corner indicate r-values and the data in the lower left corner indicate p−values.

Table S10. The t values and standard error of Pearson's correlation analysis of leaf traits of *D. odorifera* in mixed culture.

Leaf traits	LA	LT	LV	LFM	LDM	LDMC	SLA	LTD	LMF	Pn	gs	Tr	Ci	WUE	CE	LNC	PNUE	Chl a	Chl b	Chl a+b	Car
LA	−	0.551	7.013	12.116	9.673	−0.225	−0.552	0.898	0.795	0.132	−1.013	0.136	0.976	−0.260	−0.072	−0.260	0.121	0.166	−0.474	0.742	0.422
LT	0.234	−	3.089	1.392	1.082	−0.081	−2.528	−2.616	−0.115	0.540	0.114	−0.711	−0.812	1.093	0.583	−0.704	1.418	−0.227	−0.135	−0.221	−0.237
LV	0.122	0.191	−	8.771	6.347	−0.328	−1.314	−0.850	0.884	0.707	−0.728	−0.063	0.488	0.388	0.520	−0.538	0.879	0.152	−0.388	0.634	0.392
LFM	0.078	0.224	0.103	−	10.589	−0.713	−1.186	0.839	0.874	0.677	−0.946	0.013	0.293	0.245	0.515	−0.759	0.688	−0.184	−0.725	0.338	0.276
LDM	0.095	0.228	0.131	0.088	−	0.859	−2.373	1.501	0.673	−0.206	−0.967	0.247	0.653	−0.448	−0.337	−0.897	−0.166	0.145	−0.342	0.744	0.586
LDMC	0.235	0.236	0.235	0.232	0.231	−	−3.410	0.819	−0.418	−2.570	0.080	0.691	0.893	−1.964	−2.439	−0.502	−2.617	0.850	1.118	1.063	0.680
SLA	0.234	0.202	0.225	0.227	0.206	0.184	−	−0.841	−0.131	0.970	0.393	0.118	0.553	0.336	0.792	1.652	0.547	0.394	−0.070	0.154	0.088
LTD	0.231	0.201	0.231	0.231	0.222	0.231	0.231	−	−1.209	−1.189	−0.104	0.535	−0.114	−1.156	−1.095	−0.664	−1.341	−0.031	0.024	0.102	0.450
LMF	0.232	0.236	0.231	0.231	0.233	0.235	0.236	0.227	−	0.329	−1.464	−0.687	0.561	0.173	0.241	−0.228	0.050	−1.621	−2.445	−1.299	−1.472
Pn	0.236	0.234	0.232	0.233	0.235	0.202	0.230	0.227	0.235	−	−1.024	−1.414	−1.974	5.795	30.307	−0.265	6.763	0.822	−0.084	0.499	0.205
gs	0.229	0.236	0.232	0.230	0.230	0.236	0.235	0.236	0.223	0.229	−	4.031	−0.107	−1.926	−0.972	2.016	−1.486	0.797	1.661	0.893	1.410
Tr	0.236	0.232	0.236	0.236	0.235	0.233	0.236	0.234	0.233	0.224	0.171	−	0.845	−5.053	−1.518	1.997	−3.540	1.411	2.380	1.239	1.800
Ci	0.230	0.231	0.234	0.235	0.233	0.231	0.234	0.236	0.234	0.214	0.236	0.231	−	−2.199	−2.676	−0.069	−1.774	−1.001	−0.681	−0.887	−0.856
WUE	0.235	0.228	0.235	0.235	0.234	0.214	0.235	0.227	0.236	0.139	0.215	0.152	0.209	−	6.263	−1.149	11.912	−0.274	−1.081	−0.292	−0.702
CE	0.236	0.234	0.234	0.234	0.235	0.204	0.232	0.228	0.235	0.033	0.230	0.222	0.199	0.132	−	−0.348	6.562	0.796	−0.058	0.503	0.199
LNC	0.235	0.233	0.234	0.232	0.231	0.234	0.220	0.233	0.235	0.235	0.213	0.213	0.236	0.228	0.235	−	−0.376	2.767	2.415	1.853	1.898
PNUE	0.236	0.224	0.231	0.233	0.236	0.201	0.234	0.225	0.236	0.125	0.222	0.181	0.217	0.079	0.128	0.235	−	0.240	−0.580	0.171	−0.086
Chl a	0.236	0.235	0.236	0.235	0.236	0.231	0.235	0.236	0.220	0.231	0.232	0.224	0.229	0.235	0.232	0.197	0.235	−	5.309	7.432	5.110
Chl b	0.234	0.236	0.235	0.232	0.235	0.228	0.236	0.236	0.204	0.236	0.219	0.206	0.233	0.228	0.236	0.205	0.234	0.147	−	3.659	3.206
Chl a+b	0.232	0.235	0.233	0.235	0.232	0.229	0.236	0.236	0.225	0.234	0.231	0.226	0.231	0.235	0.234	0.216	0.236	0.117	0.178	−	9.752
Car	0.235	0.235	0.235	0.235	0.233	0.233	0.236	0.234	0.223	0.235	0.224	0.217	0.231	0.233	0.235	0.215	0.236	0.151	0.188	0.094	−

The data in the upper right corner indicate t-values and the data in the lower left corner indicate standard error.
